# Working horse welfare in Senegal is linked to owner’s socioeconomic status, their attitudes and belief in horse sentience

**DOI:** 10.1371/journal.pone.0309149

**Published:** 2024-10-18

**Authors:** Mactar Seck, Gemma Carder, Jennifer Wathan, Marcela Randau, Kate Fletcher, Leanne Proops

**Affiliations:** 1 Brooke Action for Horses and Donkeys, Senegal; 2 Brooke Action for Horses and Donkeys, London, United Kingdom; 3 Centre for Comparative and Evolutionary Psychology, Department of Psychology, University of Portsmouth, Portsmouth, United Kingdom; USP FZEA: Universidade de Sao Paulo Faculdade de Zootecnia e Engenharia de Alimentos, BRAZIL

## Abstract

The role that working equids play in both rural and urban communities in low and middle-income countries is invaluable. They contribute to daily tasks such as carrying food, water and people, support income generation, and are of social and cultural importance. Despite their importance, global standards of working equid welfare are low. Many variables can impact the welfare status of animals under human care, but often specific factors are explored in isolation. Factors can include, but are not limited to an owner’s socioeconomic status and their attitudes and beliefs towards animals. In this study we assessed the relationships between 1.) Attitudes and belief in horse sentience, 2.) Owner’s socioeconomic status (including household income, coverage of needs and education) and 3.) Horse welfare status. The study, consisting of an owner questionnaire and a welfare assessment of their horses, was conducted in three regions in Senegal; participants included 299 owners and their horses. Overall, our findings show that a more positive attitude towards horses, stronger belief in horse sentience, a higher standard of living and a greater ability to cover the needs of the household was associated with more positive horse welfare. A stronger belief in horse sentience was a significant predictor of horse’s body condition, larger households and those with a higher income were more likely to own a horse in good general health. Our findings demonstrate a complex relationship between working horse welfare, their owner’s attitudes, and their socioeconomic status. It is the first study we are aware of that has explored the relationships between these different variables. The findings from this study provide valuable insights into the interconnected factors which impact upon working equid welfare in Senegal and potentially more widely.

## Introduction

It is estimated that the global population of equids is 116 million (57 million horses, 50.5 million donkeys, and 7.9 million mules) [1]; Africa is considered to have the largest equid population [2]. The vast majority of the global equid population (100 million) are working animals, which provide vital contributions towards livelihoods [2, 3]. Despite the invaluable role they play, working equids are often underrepresented in national and international policies [4, 5], and are not sufficiently recognised by the United Nations’ Agenda 2030 Sustainable Development Goals [6]. Only after extensive lobbying, the United Nations formally recognised working equids as livestock in 2016 [6]. Despite their importance, global standards of working equid welfare are low, resulting in the health and welfare of many working equids being severely compromised [7]. The issues (both physical and psychological) that working equids experience can be context specific and are often dependent on the culture, local climate and the type of work they are used for [8]. Common health and welfare issues reported include poor body condition [7], musculoskeletal pathologies [9], lesions [9], dental disorders [10], behavioural issues and displays of depression-like behaviours [11]. The causal factors are multifaceted but are often linked to the exclusion of working equids in policy and international development agendas [12], socioeconomic factors such as owners inability to afford veterinary care, feed and suitable working equipment [7] and a limited knowledge base of owners [7].

The cultural, social and economic value of working equids to both rural and urban communities in low and middle-income countries (LMICs) is considerable [6, 13–16]. In many contexts they provide social status and empowerment to marginalized groups such as women [13, 15]. They support income generation across various industries including agriculture, forestry, construction, brick kilns, tourism, mining, and public transport [3] and support community resilience and recovery of communities that undergo the impacts of humanitarian crises [16]. Working equids support people from the individual household to the infrastructure of major cities [3]. Research has shown that in parts of Senegal, families produce 78% more groundnuts, 46% more maize, and 45% more millet if they have a donkey [17], and in Burkina Faso farmers would anticipate over 50% loss in most cultivated products if they were to lose their working equids [18]. In many countries equids provide a vital contribution to household tasks, such as transporting water [13], feeding livestock and reducing domestic drudgery [13]. For the benefits to both people and animals, it’s important to understand factors which influence this human-animal relationship [19].

### Relationship between working equid welfare and their owner’s socioeconomic status

The health and welfare of working equids is intimately linked to their owners [8, 20]. If animals become sick or injured and are unable to work this has a direct impact on the income generating opportunities and wellbeing of the communities who depend on them [14]. Improvements in working equid welfare significantly improve work output, productivity and efficiency [21]. In Ethiopia feed shortage and disease are noted as major constraints which effect work performance [21, 22]. Epizootic lymphangitis is an increasingly common disease that effects equids in Ethiopia, which has significant impacts on working equid owner’s socioeconomic status. This disease causes poor physical and mental welfare, and equids are sometimes abandoned due to ineffective or unavailable treatment [23]. The primary impact of reduced working capacity of equids due to this disease leads to a secondary impact on welfare as owners can then struggle to afford to buy feed for their horses and other animals [23, 24]. Likewise African Horse sickness which is endemic in many parts of sub-Saharan Africa has huge economic implications due to high mortality rates associated with the disease [25]. The economic impact largely due to losses in trade and equestrian sports is estimated to be US$95M per year [25]. Research carried out in Ethiopia found that areas which had access to animal healthcare facilities for donkeys were found to have higher animal welfare standards and owners ranked their own general well-being higher than the control area which did not have access to health-care services [26]. Owners living in areas without access to health care services for their donkeys reported feeling less confident about their income generation and the health of their animals [26]. Rough or aggressive handling has also been shown to substantially reduce productivity of working equids [7]. In relation to other common health and welfare issues associated with working equids, notable reductions in income have been reported by communities. Owners have reported a reduced income caused by equid foot disorders in Kenya [27] and Ethiopia [28]. A recent review paper discusses the detrimental effects of poor health and welfare of working equids on women [29] some of these consequences include impacts on their health and social status [29]. For example, in some LMICs women who lose their equids, or have a sick of injured one may resort to prostitution to support their families [30].

In comparison, how the socio-economic status of owners can impact health and welfare of working equids is less clearly understood, there appears to be only two published studies that have explored this. The first study conducted in Gwalior, India compared equid welfare and socio-economic status of three groups 1). Equids working at brick-kiln sites; 2). Equids working in stone quarries, and 3). Equids working at city sites. The socio-economic status (measured by income and school-going children) of group 1 owners and the welfare of their equids was found to be comparatively poorer than that of the other two groups [31]. This relationship was attributed to owner’s skills, knowledge, husbandry practices and attitude to how they work their equids (e.g. loads and working period) being lower with a lower socio-economic status, meaning that equids were forced to work for longer hours and were therefore overworked [32]. A more recent study conducted in Chile has contrasting findings, demonstrating that socio-economic status had no impact on the welfare of working equids. Of the 72 horse owners interviewed, 91.4% of them were considered vulnerable according to the vulnerability index measurement, yet only 28.3% of equids were found to be in a poor welfare state [33]. The contrast in findings are likely due to the different contexts and different methods used. A further important consideration of such findings is that psychological factors such as empathy, compassion and attitudes and beliefs towards animals are likely to also effect animal health and welfare [20]. The relationship between such factors and socioeconomic status appears to be less understood.

### Relationship between owner’s compassion, attitudes, belief in animal mind and animal welfare

Empathy refers to our general capacity to resonate with others’ emotional states (both positive and negative) [34]. Empathy towards animals has been linked to current pet ownership and ownership of pets during childhood (in High Income Countries (HICs)) [35]. The inability to experience empathy has been linked to anti-social behaviour including animal cruelty [36]. Compassion not only involves the feeling of being effected by a person’s suffering, but also wanting to act to help them [34]. Five components have been proposed 1). Recognising suffering; 2). Feeling empathy for the person suffering and connecting with the distress; 3). Tolerating uncomfortable feelings aroused in response to the suffering person; 4). Understanding the universality of suffering in the human experience and 5). Acting or being motivated to act to alleviate suffering [34].

Research findings have demonstrated a link between empathy and animal welfare. Lanas et al. [33] reported that higher levels of empathy and equine pain perception in owners were correlated with better equid welfare. Kielland et al. [37] found that farmers who scored more highly for empathy owned cattle with less skin lesions. Published work on compassion has focussed primarily on compassion towards humans. One exception is a study which developed the Children’s Compassion toward Animals (CCA) measure [38]. Findings indicate higher levels of Belief in Animal Mind (BAM) in children was associated with compassion towards pets. BAM involves attributing animals with mental capacities such as intelligence and feelings of emotions [39]. A number of studies in different contexts and with different species have shown an association between BAM and positive/negative attitudes and positive/negative animal welfare. For example, research has shown a relationship between higher levels of empathy and higher perception of pain in working equids and an overall better equid welfare state [33]; recognition of emotion by owners and better donkey welfare [40]; stockpersons who express pleasure from working with pigs and feeling of empathy towards them owned less fearful pigs [41]; and a relationship between farmer’s Belief in Animal Mind and positive attitudes towards dairy cattle and positive animal welfare [37].

Attitudes can be a predictor of behaviour [42, 43], therefore understanding people’s attitudes towards animals is important for animal welfare interventions to be successful. A person’s attitude towards animals may affect how they react around them, therefore effecting the quality of the human-animal interaction [42]. A recent study found that working equid owners who believed their animals could feel emotions owned equids that had a better health status and body condition, compared to owners who believed their animals could not feel emotions [44]. Similar findings showing a relationship between BAM and positive animal welfare have been found in pert rabbits [45], pigs [46] and dairy cows [47]. In turn, people’s attitudes towards animals and belief in animal minds can be affected by different factors including their gender [48, 49], cultural background [50] familiarity with animals [51], and the species in question [52].

The impact that animal owner’s socioeconomic status has on their animal’s welfare is under researched, and studies that have explored links between attitudes to animals (including BAM) and animal welfare has primarily been conducted in high-income countries (HICs). Furthermore, there appears to be no published literature which investigates the relationship between socioeconomic status, animal welfare and attitudes and beliefs towards animals in a single study. This study therefore aimed to explore relationships between these variables in a lower-middle income country, Senegal.

## Methods

### Ethics

Brooke’s Animal Welfare Ethical Review Body and the University of Portsmouth’s Ethics Committee for the Faculty of Science and Health and the Animal Welfare Ethical Review Board reviewed and approved the study design (Ref: SFEC 2018-121B; 1018G).

### Study location and population

Data collection took place during July and August 2022. The participants included in this study were from towns and villages in the Senegalese regions Diourbel, Louga and Thiès. Selection criteria for participants were 1.) Head of household; 2.) Horse owners and users 3.) Willingness to participate in the study and 4.) over the age of 18 years. In total 299 heads of household participated in the study (males N = 276, females N = 23). The females were all widows or emigrant wives, and de facto heads of families. The mean age of participants (excluding 5 people whose ages were not provided) was 54.65 years (range = 18–85, SD = 14.93 years). The study included 79 female and 220 ungelded male horses with a mean age of 8.10 years (range = 1–30, SD = 5.73 years), excluding 10 whose ages were unknown. Most horses (N = 269, 90%) were used for agriculture, with the remaining being used for transporting goods (n = 25, 8%) and people by cart (n = 2, 1%), and two were foals, which were not yet used for work.

### Tools

For the purpose of this study, research tools that had previously been validated were adapted (with the exception of the welfare assessment tool). The tools were used to assess the following 1.) Compassion towards horses 2.) Attitudes towards horses and belief in horse minds, 3.) Owner’s socioeconomic status (including household income, coverage of needs and education) 4.) Horse welfare status.

### Compassion for horses scale

The previously validated Sussex-Oxford Compassion for Others Scale (SOCS-O) method of measuring compassion towards other people [53] was adapted for the purpose of assessing compassion towards horses. The SOCS-O is specifically designed to measure compassion as defined by Strauss et al (2016). The design of the SOCS-O questionnaire allowed for modification by replacing reference to people with reference to the owner’s horses, for example ‘when others are struggling, I try to do things that are helpful’, ‘when my horse(s) are struggling, I try to do things that would be helpful’ [see **S1 Table** for details].

### Horse Attitude and Beliefs Scales

Assessment of attitudes and beliefs using a series of statements were adapted from two models/scales. These were the four-item Belief in Animal Minds Scale (BAMS) [54]; which measures participants’ attributions of mental states, emotions and other abilities to animals (for the purpose of this study ‘animal’ was replaced with ‘horse’) and the Human-Animal Relationship model [55] which is based on the theory of planned behaviour [56], which states that three key drivers impact on stockperson behaviour 1.) Attitudes towards the behaviour, 2.) Subjective norms and 3.) Perceived behavioural control. Herein referred to as the Horse Attitude Scale (HAS). The models were adapted to be used with working horse owners and within the Senegalese context [see **S2 Table** for details].

### Horse owner’s socioeconomic status

Horse owner’s socioeconomic status was assessed using a combination of the simplified balance sheet of the family farm which is widely used by human development organisations in West Africa [57] and dimensions from the Household Questionnaire within the Multidimensional Poverty Index, designed to measure acute poverty across developing countries [58]. We adapted the simplified balance sheet of the family farm into a seven-point Coverage of Needs Scale (CoNS) documenting the extent to which the household income covered the needs of the family. The scale ranged from “family income does not cover the family needs and the family has high debts and is unable to pay them in due time”, through “family income covers needs but the family is unable to save” to “family income exceeds needs and the family can regularly save so that extra expenses are supported whenever necessary”. From the Multidimensional Poverty Index, we used the Standard of Living Index (SLI) as a measure of economic status. These questions determined the extent to which the family owns key items such as a refrigerator, phone, motorised transport, and whether their home is made of suitable material. We also included household size (Size) and household income (Income). To assess potential educational factors, the literacy of head of household (Literacy: Yes/No), whether any household member had completed 6 years of education (Six years: Yes/No) and whether all school-aged children were enrolled in school (Enrolled: Yes/No) were also taken from the Poverty Index [**S3 Table**].

### Horse welfare assessment

For the purposes of this study the Primary Assessment of Welfare Experiences’ (PAWE) tool was developed [**S4 Table**]. The non-intrusive tool was designed to assess working equid (horse, donkey or mule) welfare, without being time or resource intensive. The tool is based on the 2020 five domains model [59] and is used to determine whether the welfare of an equid in relation to a specific domain is unacceptable, acceptable or good. Each of the four physical domains (nutrition, environment, health and behaviour) were outlined, with relevant emotional experiences outlined under each of these, to try to encourage emotional state to be considered when assessing each individual domain rather than seeing it as a ‘separate’ domain. Within each domain, positive indicators of welfare were scored independently of negative indicators of welfare to create a positive and a negative welfare score. Assessment of the degree in which an animal experienced each emotion was based on Mendl et al (2010) [60] discussion of high or low arousal and positive and negative valence. Guidance notes were given for each emotion within each domain to prompt assessors to consider variables which could affect the animal’s experience.

Selected measures from the Standardised Equine-Based Welfare Assessment Tool (SEBWAT) [61] were also included [**S4 Table**]. These measures were body condition score (BCS), gait, and lesions. The data collectors provided a holistic view of the general health status (GHS) of the horses.

All seven data collectors were previously certified in SEBWAT and underwent training and a standardization process to minimize scoring differences. The tool was trialled and validated by data collectors scoring a minimum of 15 animals each from the same or equivalent areas as those included in the study. Scores were then compared for reliability and calibration. The intra-class correlation was excellent, ICC = .97 (.965 - .975 CI).

### Procedure

Interviews and animal welfare assessments took place at the participant’s home. Prior to data collection all assessors were trained on the protocol. The interviews using the Horse Attitude and Belief Scales and the Modified Sussex-Oxford compassion for others scale and PAWE were conducted by two assessors. An additional 12 assessors were involved in the collection of the socioeconomic status data. The questionnaires were written in English and later translated into French, the data collectors were native French speakers who interviewed the study participants in the local language (Wolof). Translations were done accurately, however it was also important to use terms and expressions that could be easily understood by the participants and were context specific (pers comms, 2024). There was a high illiteracy rate amongst participants, therefore the enumerators verbally explained the study to the participants and obtained verbal informed consent. All data were collected verbally and recorded simultaneously using printed questionnaires. For owners who had multiple horses, one horse per household was selected to take part, this was the horse which was used for work (usually a stallion). None of the households owned more than one stallion, therefore the selection method remained consistent throughout the data collection period. The welfare assessments using PAWE were done first, each horse was unloaded and unharnessed when the assessments were made, assessments were made at home. The horses were initially observed from a distance while assessments in regards to nutrition, environment and health were made and recorded. Some of PAWE’s behaviour descriptors required assessment in response to the human/owner approach. These were labelled “HA” and were completed at the end of the PAWE assessment. For each descriptor a cross was marked on a scale, representing a minimum to maximum score (see **S4 Table**). Data collectors also made notes for each descriptor. Following the use of PAWE, assessments were made using the following three SEBWAT indicators: body condition score (BCS); horses could be scored between 1–4 (1 = extremely thin, 4 = an ideal weight), gait, and lesions, plus a holistic indication of general health status. The welfare assessment took approximately fifteen minutes per horse to complete.

Subsequently, owners were verbally asked a series of questions from the Horse Attitude and Beliefs Scale followed by the Modified Sussex-Oxford Compassion for Others Scale. They were read a series of statements such as “If I treat my horse well it improves its quality of life.” Each participant was asked “How do you feel about that? Do you agree, disagree, or don’t know/both agree and disagree?”. During a second step they were asked if they strongly agree or strongly disagree. The data collectors marked the most appropriate box on the questionnaire. Finally, the socioeconomic status of households was assessed using the pre-determined questionnaire. The three questionnaires combined took forty-five minutes per participant to complete.

Hard copies of the data collection sheets were securely stored, the participant’s personal details such as name and address were not recoded. One researcher (who did not participate in the data collection) entered the data into a Microsoft Excel spreadsheet for later analysis.

### Data analysis

The attitudinal and socioeconomic factors outlined above (Attitudinal: Horse Attitude Scale (HAS), Belief in Animal (Horse) Mind (BAM); Socio-economic Status: Coverage of Needs Scale (CoNS), Standard of Living Index (SLI), Household Size, Household Income, Literacy of Head of Household, Six years of Education, Children enrolled in school) were included as predictors of the five welfare outcome variables (PAWE-Positive, PAWE-Negative, Body Condition Score (BCS), Lesions and General Health Status GHS)) in a series of GLMM models in R Studio [62] using the glmm TMB package [63] for continuous (PAWE-Pos, PAWE-Neg) and binomial (Lesions, GHS) variables and the ordinal package for BCS [64]. The random factor Subject ID nested within Village was included in the models continuous and binomial models. The ordinal models failed to converge with a nested random factor and so Village alone was included. Initial data exploration of predictor and outcome variables revealed that the responses from the Compassion Scale showed a significant ceiling effect, with a mean score of 98.99 (±1.70) out of 100, with 87% of participants scoring 98% or above. We therefore excluded the Compassion Scale from further analyses. The outcome variable Gait also showed little variation in scores, with 284 horses (95%) recorded as showing no gait abnormalities, 14 showing minor abnormalities and 1 showing significant abnormalities. We therefore also excluded the outcome measure Gait from further analyses. The variable PAWE-Neg showed strong positive skew and was log transformed.

The predictor variables BAM and HAS were collinear (with a correlation coefficient greater than 0.4) and were excluded from the same models (N = 283, r = .70, P<0.0001). A series of candidate models were run for each outcome variable including the null, global (with BAM), global (with HAS). Factors with little or no predictive value were systematically removed from the global models to reach a best fit model. Akaike’s Information Criterion adjusted for small sample size (AICc) was used to rank the models and the significance of each predictor variable was derived from the best fit model.

## Results

### Body condition score (BCS)

**Fig 1:**
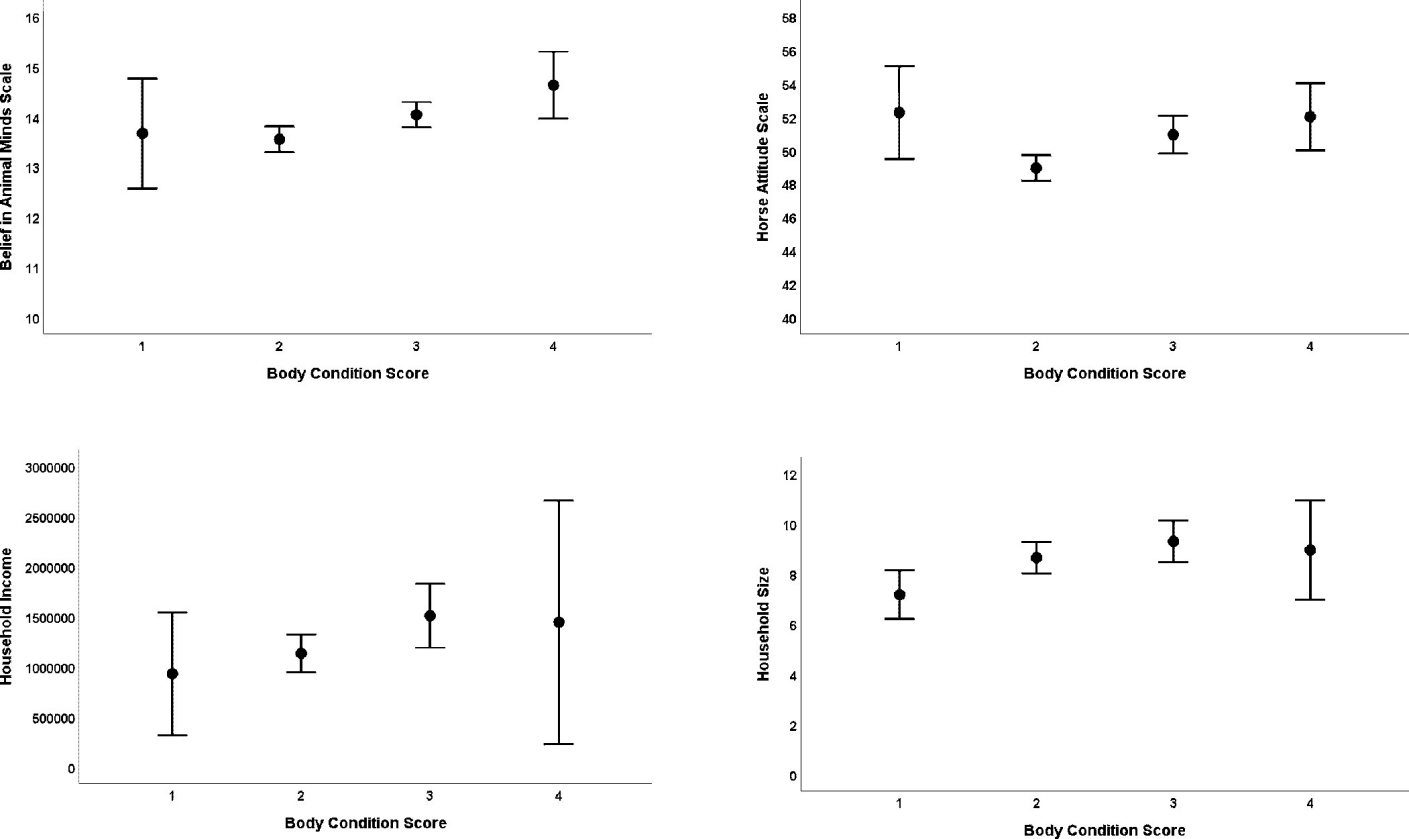
Error bar plots of the raw data from the significant predictor variables in the best fit model for the welfare outcome measure body condition score. Error bars represent 95% confidence intervals.

The mean BCS for the horses was 2.48 ±. 068 (range = 1–4), with 12 (4%) horses scoring 1, 150 (50%) horses scoring 2; 118 (40%) horses scoring 3, and 19 (6%) horses scoring 4. The best fit model contained the predictor variables Belief in Animal (Horse) Minds (z = 3.6, p < .001), Household Size (z = 2.3, p = .02) and Income (z = 1.9, p = .05) **(Fig 1)**. Specifically, owners that possessed a stronger BAM, those with large households and those with a greater income owned horses with a higher BCS. Although the HAS could not be included in the same model as BAMS due to a high level of collinearity, in a single factor model, a more positive attitude towards horses was also significantly predictive of a higher BCS (z = 2.6, p = .009). No other predictor variables were associated with BCS.

### PAWE-Positive

**Fig 2:**
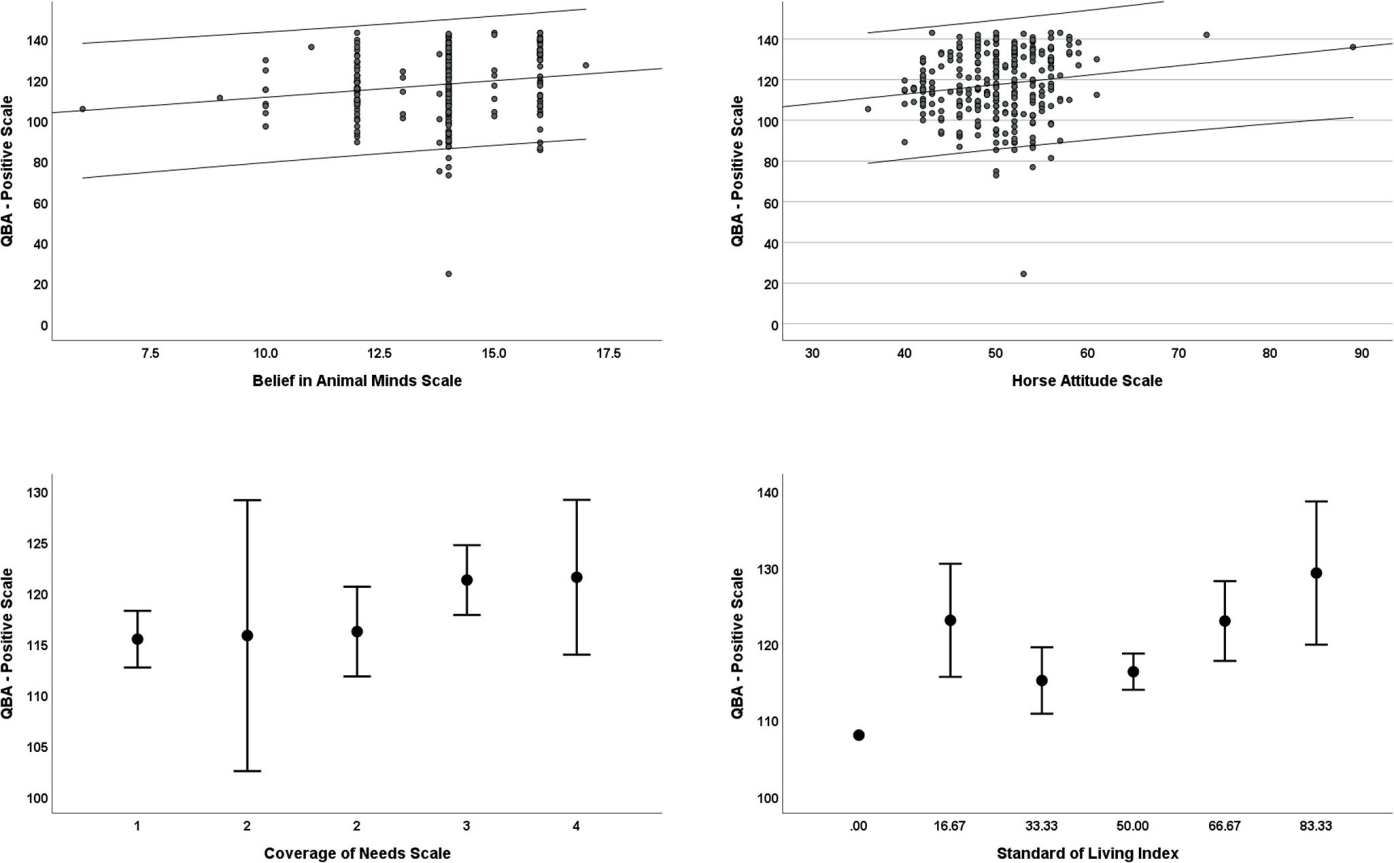
Error bar and scatter plots of the raw data from the predictor variables in the best fit model for the welfare outcome measure PAWE-Positive. Error bars and outer regression lines represent 95% confidence intervals.

The mean score for the positive element of the PAWE was 117.52 ± 16.25 (Mean ± SD; range = 25–143). The best fit model contained the predictor variables HAS (z = 2.4, p = .02), CoNs (z = 1.9, p = .06) and SLI (z = 1.7, p = .09) **(Fig 2)**. Specifically, a more positive attitude towards horses, a higher standard of living and a greater ability to cover the needs of the household were associated with higher scores on the PAWE-Positive. Although the BAMS could not be included in the same model as the HAS due to a high level of collinearity, in a single factor model, a stronger belief in horse sentience was also significantly predictive of higher PAWE Positive score (z = 2.3, p = .02). No other predictor variables were associated with the PAWE-Positive score.

Within the PAWE-Positive subscales, BAM was positively correlated with Positive Nutrition (r = .30, p = < .001), Positive Health (r = .13, p = .03) and Positive Behaviour (r = .19, p = .001) but not with a Positive Environment (r = .09, p = .12). Higher (more positive) scores on the HAS was positively associated with Positive Nutrition (r = .21, p = < .001) and Positive Behaviour (r = .18, p = .003) but not with Positive Environment (r = .04, p = .53) nor Positive Health (r = .04, p = .53). CoNS positively correlated with all subscales: Positive Nutrition (r = .13, p = .03); Positive Environment: r = 0.12, p = .05); Positive Health (r = .17, p = .005) and Positive Behaviour (r = .13, p = 0.03). The Standard of Living Index positively correlated with Positive Nutrition (r = .20, p = < .001) and Positive Health (r = .13, p = .04) but not with Positive Environment (r = .01, p = .87) nor Positive Behaviour (r = -.10, p = .10) **(Fig 2)**.

### PAWE-Negative

**Fig 3:**
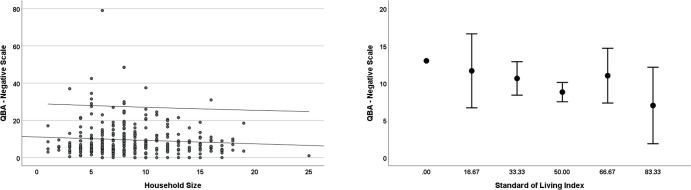
Error bar and scatter plots of the raw data from the predictor variables in the best fit model for the welfare outcome measure PAWE-Negative. Error bars and outer regression lines represent 95% confidence intervals.

The mean score for the negative element of the PAWE was 9.46 ± 9.04 (range = 0–79). The best fit model contained the predictor variables Standard of Living (z = -1.8, p = .07) and Household Size (z = -1.9, p = .06). Specifically, the lower the standard of living, and the smaller the household size, the higher the PAWE-Negative score and the worse the horse welfare (**Fig 3**). Within the PAWE-Negative subscales, Standard of Living was correlated with Negative Environment (r = .14, p = .02) but not with Negative Nutrition (r = .10, p = .08), Negative Health (r = .01, p = .81) nor Negative Behaviour (r = -.07, p = .25). Household Size was correlated with Negative Nutrition (r = -.13, p = .03) but not with Negative Behaviour (r = .03, p = .61), Negative Environment (r = -.08, p = .18) nor Negative Health (r = .01, p = .82). None of the attitude or education variables predicted the negative subscales of PAWE.

### General health status (GHS)

**Fig 4:**
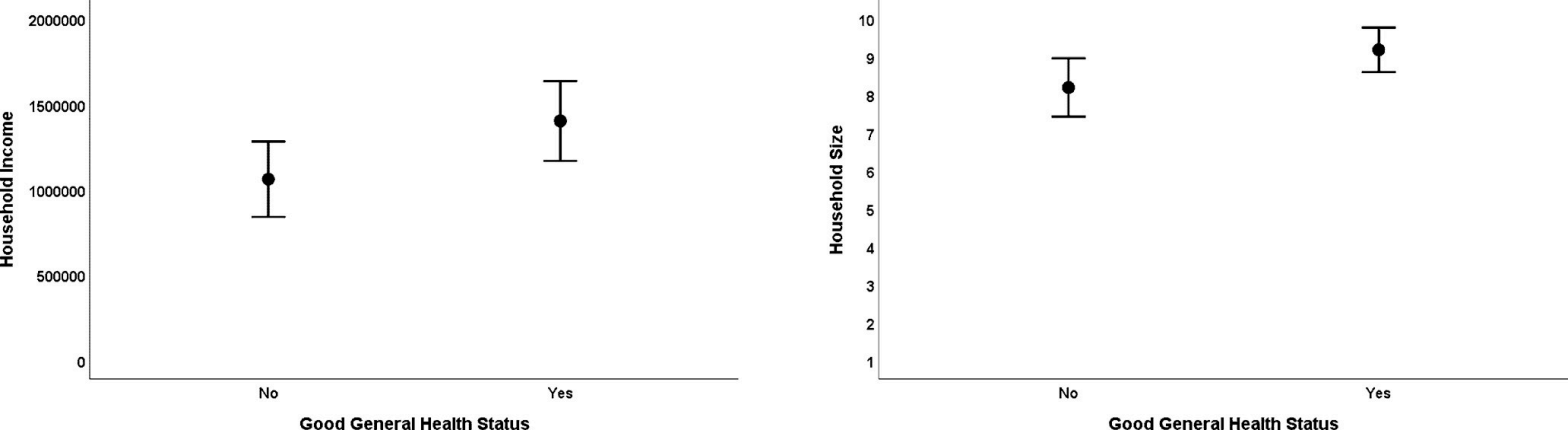
Error bar plots of the raw data from the significant predictor variables in the best fit model for the welfare outcome measure general health status. Error bars represent 95% confidence intervals.

The majority of horses were thought to have a good GHS (203 horses/ 68%), with the health status of 81 (27%) horses scored as fair and 15 (5%) as poor. The horses scored as having fair or poor health status were combined into a group of horses that have “compromised general health” for the purposes of statistical analysis. The best fit model contained the predictor variables Household Size (z = -1.8, p = .08) and Income (z = 1.6, p = .11). Specifically larger households and those with a higher income were more likely to own a horse in good general health **(Fig 4)**.

### Lesions

The majority of horses did not show any lesions (253 horses; 85%), with 46 horses showing evidence of some lesions. None of the predictor variables significantly predicted the likelihood of a horse having lesions, with the null model being the best fit.

## Discussion

The results from this study offer an initial understanding of the relationship between working horse welfare, owner’s attitudes and beliefs, and their socio-economic status. As far as we are aware this is the only study to date which has explored these interconnections. The findings from this study highlight the importance of understanding the relationship between socioeconomic status, attitudes, belief in animal mind, and animal welfare. Studying these variables in isolation, doesn’t allow for the ‘complete picture’ to be understood, therefore understanding the complex dynamics between the variables is invaluable.

Horse welfare assessments were made using three indicators from SEBWAT (body condition score, gait and lesions) and the use of PAWE. Our findings show that owners who possess a stronger belief in horse sentience and have a more positive attitude towards their horses own horses who have a higher (healthier) BCS. This supports findings from a recent cross-cultural study which found that Senegalese horse, donkey and mule owners who believed their animals could feel emotions owned horses with a higher BCS compared to owners who thought they couldn’t [44] and a study in Pakistan where an association between recognition of donkey emotions and a better welfare status was reported [40]. BAM was positively correlated with positive nutrition, health and behaviour, similarly higher scores on the HAS was associated with positive nutrition and behaviour, however it was not associated with positive environment or health. Different factors such as age, gender and culture can influence our attitudes towards other species [51], it is also suggested that BAM is a strong determinant of attitudes towards other species [54]. Therefore, it is understandable that both BAM and more positive attitudes towards horses were both associated with positive elements of PAWE, although it’s worth noting that BAM was associated with better horse health, but higher HAS scores were not.

We did not find an association between BAM and attitudes towards horses with the positive environment subscale of PAWE, however owner’s coverage of needs and the Standard of Living Index did positively correlate with positive environment scores. The coverage of needs scale aimed to document the extent to which the household income covered the needs of the individual families and the Standard of Living Index (SLI) was used to assess economic status. It is possible that belief in horse mind and positive attitudes to horses (within this context) are not enough in isolation to provide positive environmental conditions such as shelter, a suitable resting area, adequate space and appropriate social conditions. Thus, factors in relation to the economic status of the horse owners and their families are a better predictor of the provision of a welfare friendly environment.

Coverage of needs correlated with all the positive subscales of PAWE (nutrition, environment, health and behaviour). Therefore, within this context if the family’s needs were covered for instance if they could pay their bills and/or they could afford to put savings aside they would invest in their horse’s physical welfare. For example, households would provide an appropriate diet, suitable housing and veterinary care. It’s important to note however, that catering for animal’s physical needs in isolation does not result in overall good welfare. Similarly, households with a higher income were more likely to own a horse in good general health; also larger households more likely to own a horse in good general health. An explanation for this, could be that in addition to a higher income, families are more resilient and a larger family results in more carers for the horses(s). Interestingly, the Standard of Living Index positively correlated with positive nutrition and positive health, but not positive behaviour or environment. Perhaps positive behaviour and environment are associated and effected by additional factors, for example a positive environment may be largely influenced by the availability of resources.

In contrast to the positive subscales of PAWE, none of the attitude or education variables predicted the negative subscales of PAWE, and only two economic factors weakly predicted them. Relative to the welfare of working horses outlined in other studies conducted in LIMCs [7, 65, 66], the body condition and general health status of this population was fairly good overall. The scores on the negative subscales of PAWE also strongly clustered at the lower end of the scale. It may well be that this lack of variation in negative welfare scores meant that the predictor variables lacked discriminative ability. It would be interesting to explore this hypothesis in other working equid populations in LIMC’s where welfare was lower and also more varied.

No association was found between formal education level and horse welfare. This is not surprising, animal management knowledge, which is usually gained from family and community members is likely to be a more important contributor to good horse welfare than number of years at school or the education level of horse owners.

Due to a celling effect the data collected using the Compassion for Horses Scale was removed from this study, there are two possible explanations for this. Firstly, it may be due to the adaptations we made to the original Sussex-Oxford Compassion for Others Scale; perhaps it was not discriminative enough to be used with animals, and secondly it may be related to the Senegalese context. Haddy et al (2023) found a celling effect in owner response to belief in working equid emotions and pain in Pakistan; it is suggested that this could be a result of culture-level acquiescent bias. It is also worth noting that response bias may have affected the results, data for this study was collected on behalf of international Non-Governmental Organisation- Brooke Action for Working Horses and Donkeys (Brooke), and therefore it is possible that participants wanted to give a ‘positive’ response. Although in many cases participants were not familiar with the work of Brooke.

### Considerations for future study

In this study there was an imbalance between the number of male and female participants, with the majority of participants being male. Gender bias has also been reported in research concerning working horses in Ethiopia [23]. The reason for the gender imbalance in this study, is that within the context that the study took place heads of households are usually male. Future studies would benefit from a more balanced gender inclusion of participants, attempts could be made to recruit female members of the household to participate. This could be valuable as females within Senegal and other low- and middle-income countries are often the carers of their animals [15], therefore understanding their view point is hugely valuable. Furthermore gender is believed to contribute towards some of the variance in Belief in Animal Mind and attitudes towards animals [67]; compared to females, males are believed to present lower levels of Belief in Animal Mind [67]. It would also be beneficial to include participants with a variety of different roles within the household to further understand how BAM, attitudes and socioeconomic status impact on animal welfare.

Participants included in this study were over the age of 18. Future studies within Senegal and other LMICs could explore the relationship between children’s Belief in Animal Mind, attitudes and working horse welfare. A study conducted in the UK, found that child belief in animal mind was associated with a higher attachment to pets, and humane and caring behaviour towards animals [38], this could be explored within the Senegalese context in relation to working animals. This could be a valuable insight, particularly if children within the household are involved in caring for the animals.

This study was focused solely on horses. Future studies could also explore differences in belief in animal mind, attitudes and animal welfare between different working animal species used within Senegal. It would be interesting to know if the same associations between belief in animal mind, attitudes and socioeconomic status also occur in relation to other animal species welfare. Such knowledge could help inform education programmes targeted at communities who own different species of working animal, and in particular species such as donkeys which in some contexts are viewed negatively (personal communication, 2024). Future studies would also support understanding this area better in LMICs, as to date most research has been conducted in HICs. Future studies could also explore a reliable compassion for animals’ scale.

Working horses are used across the globe for a variety of work types, they often face a variety of welfare challenges. Understanding how their owner’s beliefs, attitudes and socioeconomic status may contribute towards their welfare status is hugely important for programmatic interventions aimed at improving animal welfare, and data is essential to effectively advocate for policy changes. It is important to note that in addition to the impact that socioeconomic status, attitudes and beliefs have on animal welfare, an animal’s welfare status can also impact upon household’s socioeconomic status. This relation could be explored further in future studies. It is anticipated that the data generated will support Brooke West Africa’s donors and partnership engagement plan, communication and advocacy initiatives and raise awareness about the importance of horses in Senegal. The findings can be used to support advocacy initiatives targeted at authorities to implement effective measures to protect the horses who play vital roles in people’s livelihoods; convince donors to fund equine welfare projects; and provide an evidence base to convince other development INGOs to engage in joint projects aiming to improve both human and equine welfare. Information will be provided to the communities involved in the study to raise awareness on the interdependency between their own wellbeing and the welfare of their horses. We hope that our approach and the findings generated from this study inspire future research in this area in other countries.

## Conclusion

Our findings demonstrate a complex relationship between working horse welfare, their owner’s attitudes and belief in horse mind, and their socioeconomic status. It is the first study we are aware of that has explored the relationships between these different variables. Understanding the drivers that contribute towards ensuring good animal welfare are critical for those working to improve the lives of animals. This study provides an initial insight which can be used to inform future research and can be used by INGOs for evidence-based programming and advocacy.

## Supporting information

S1 Table Modified Sussex-Oxford Compassion for Others Scale. (PDF)

S2 Table Attitudes and Beliefs Scale. (PDF)

S3 Table Socioeconomic status and coverage of needs tools. (PDF)

S4 Table Animal welfare assessment: Primary assessment of welfare experiences tool and Standardised Equine Based Welfare Assessment Tool (measures used). (PDF)

S1 Data Full data sheet. (XLSX)

S1 File Inclusivity in global research form. (DOCX)
