# Supplementary material for: Working horse welfare in Senegal is linked to owner’s socioeconomic status, their attitudes and belief in horse sentience
Source: PLoS One. 2024 Oct 18;19(10):e0309149. doi: 10.1371/journal.pone.0309149 (PMC11488707; doi:10.1371/journal.pone.0309149)
Supplement: S1 Table — (PDF) [file pone.0309149.s001.pdf]

S1 Table. Modified Sussex-Oxford Compassion for Others Scale (SOCS-O)

<table>
<tr><th>Statement</th><th colspan="5">Participant’s answer</th><th>Notes</th></tr>
<tr><td rowspan="2">I recognise when horses are feeling distressed.* (1)</td><td colspan="2">Agree</td><td>Don’t know or mixed feelings</td><td colspan="2">Disagree</td><td rowspan="2"></td></tr>
<tr><td>A lot</td><td>A little</td><td>n/a</td><td>A lot</td><td>A little</td></tr>
<tr><td rowspan="2">I understand that like every living being, horses experience suffering at some point in their lives. α (2)</td><td colspan="2">Agree</td><td>Don’t know or mixed feelings</td><td colspan="2">Disagree</td><td rowspan="2"></td></tr>
<tr><td>A lot</td><td>A little</td><td>n/a</td><td>A lot</td><td>A little</td></tr>
<tr><td rowspan="2">When a horse is going through a difficult time, I feel kindly towards them.β (3).</td><td colspan="2">Agree</td><td>Don’t know or mixed feelings</td><td colspan="2">Disagree</td><td rowspan="2"></td></tr>
<tr><td>A lot</td><td>A little</td><td>n/a</td><td>A lot</td><td>A little</td></tr>
<tr><td rowspan="2">When a horse is upset, I try to understand and accept their feelings rather than avoid or ignore them. ∞ (4)</td><td colspan="2">Agree</td><td>Don’t know or mixed feelings</td><td colspan="2">Disagree</td><td rowspan="2"></td></tr>
<tr><td>A lot</td><td>A little</td><td>n/a</td><td>A lot</td><td>A little</td></tr>
<tr><td rowspan="2">When horses are struggling, I try to do things that would be helpful.[π] (5)</td><td colspan="2">Agree</td><td>Don’t know or mixed feelings</td><td colspan="2">Disagree</td><td rowspan="2"></td></tr>
<tr><td>A lot</td><td>A little</td><td>n/a</td><td>A lot</td><td>A little</td></tr>
<tr><td rowspan="2">I notice when my horses are feeling distressed.* (6)</td><td colspan="2">Agree</td><td>Don’t know or mixed feelings</td><td colspan="2">Disagree</td><td rowspan="2"></td></tr>
<tr><td>A lot</td><td>A little</td><td>n/a</td><td>A lot</td><td>A little</td></tr>
<tr><td rowspan="2">I understand that feeling upset at times is part of a horse’s nature.[α](7)</td><td colspan="2">Agree</td><td>Don’t know or mixed feelings</td><td colspan="2">Disagree</td><td rowspan="2"></td></tr>
<tr><td>A lot</td><td>A little</td><td>n/a</td><td>A lot</td><td>A little</td></tr>
</table>

Statement	Participant's answer					Notes
When I hear about bad things happening to horses, I feel concern for their wellbeing.[β](8)	Agree		Don't know or mixed feelings	Disagree		
	A lot	A little	n/a	A lot	A little	
I stay with horses when they're upset even if it's hard to bear.[∞] (9)	Agree		Don't know or mixed feelings	Disagree		
	A lot	A little	n/a	A lot	A little	
When a horse is going through a difficult time, I try to look after them.[π] (10)	Agree		Don't know or mixed feelings	Disagree		
	A lot	A little	n/a	A lot	A little	
I'm quick to notice early signs of distress in horses.* (11)	Agree		Don't know or mixed feelings	Disagree		
	A lot	A little	n/a	A lot	A little	
Like me, I know that horses also experience struggles in life.[α](12)	Agree		Don't know or mixed feelings	Disagree		
	A lot	A little	n/a	A lot	A little	
When a horse is upset, I try to understand how they're feeling. [β](13)	Agree		Don't know or mixed feelings	Disagree		

	A lot	A little	n/a	A lot	A little	
I connect with the suffering of horses without judging them. [∞] (14)	Agree		Don't know or mixed feelings	Disagree		
	A lot	A little	n/a	A lot	A little	
When I see a horse in need, I try to do what's best for them. [π] (15)	Agree		Don't know or mixed feelings	Disagree		
	A lot	A little	n/a	A lot	A little	
I recognise signs of suffering in horses.* (16)	Agree		Don't know or mixed feelings	Disagree		
	A lot	A little	n/a	A lot	A little	
**Statement**	**Participant's answer**					**Notes**
I know that we can all feel upset at times, including horses, when bad things happen to us. [α](17)	Agree		Don't know or mixed feelings	Disagree		
	A lot	A little	n/a	A lot	A little	
I'm sensitive to horse's distress. [β] (18)	Agree		Don't know or mixed feelings	Disagree		
	A lot	A little	n/a	A lot	A little	
When a horse is upset, I can be there for them without feeling overwhelmed by their distress. [∞] (19)	Agree		Don't know or mixed feelings	Disagree		
	A lot	A little	n/a	A lot	A little	
When I see that my horse is upset, I do my best to take care of them. [π] (20)	Agree		Don't know or mixed feelings	Disagree		
	A lot	A little	n/a	A lot	A little	

Recognising suffering* 1, 6, 11, 16.
Understanding the universality of suffering[α] 2, 7, 12, 17.
Feeling for the horse suffering[β] 3, 8, 13, 18.
Tolerating uncomfortable feelings[∞] 4, 9, 14, 19.
Acting or being motivated to act to alleviate suffering[π] 5, 10, 15, 20.
