# Supplementary material for: Working horse welfare in Senegal is linked to owner’s socioeconomic status, their attitudes and belief in horse sentience
Source: PLoS One. 2024 Oct 18;19(10):e0309149. doi: 10.1371/journal.pone.0309149 (PMC11488707; doi:10.1371/journal.pone.0309149)
Supplement: S2 Table — (PDF) [file pone.0309149.s002.pdf]

S2 Table. Attitudes and beliefs scale

Statement	Participant's answer					Notes
If I treat my horse well it improves its quality of life.* (1)	Agree		Don't know or mixed feelings	Disagree		
	A lot	A little	n/a	A lot	A little	
It is important to me that other people think my horse looks clean and well groomed. α (2)	Agree		Don't know or mixed feelings	Disagree		
	A lot	A little	n/a	A lot	A little	
It is difficult for me to allow my horse to have time to rest during working hours.β (3).	Agree		Don't know or mixed feelings	Disagree		
	A lot	A little	n/a	A lot	A little	
Horses are unaware of what is happening to them. ∞ (4)	Agree		Don't know or mixed feelings	Disagree		
	A lot	A little	n/a	A lot	A little	
Regularly grooming my horse is beneficial to his general health.* (5)	Agree		Don't know or mixed feelings	Disagree		
	A lot	A little	n/a	A lot	A little	
My family disapproves if I offer my horse rest during working hours.α (6)	Agree		Don't know or mixed feelings	Disagree		
	A lot	A little	n/a	A lot	A little	
No matter how much I groom my horse it will still have dirt and ectoparasites β (7)	Agree		Don't know or mixed feelings	Disagree		
	A lot	A little	n/a	A lot	A little	
Horses are capable of experiencing a range of feelings and emotions (e.g. pain, fear, contentment, maternal affection).∞(8)	Agree		Don't know or mixed feelings	Disagree		
	A lot	A little	n/a	A lot	A little	

Statement	Participant’s answer					Notes
Giving proper rest to my horse during working hours will result in a longer working life.* (9)	Agree		Don’t know or mixed feelings	Disagree		
	A lot	A little	n/a	A lot	A little	
Most of my neighbours think it is normal to keep a horse alone and separate from other horses. α (10)	Agree		Don’t know or mixed feelings	Disagree		
	A lot	A little	n/a	A lot	A little	
Even if I handle my horse compassionately, it will still be aggressive. β (11)	Agree		Don’t know or mixed feelings	Disagree		
	A lot	A little	n/a	A lot	A little	
Horses are beings who feel pain. ∞ (12)	Agree		Don’t know or mixed feelings	Disagree		
	A lot	A little	n/a	A lot	A little	
Allowing horses to live with other horses will improve their welfare.* (13)	Agree		Don’t know or mixed feelings	Disagree		
	A lot	A little	n/a	A lot	A little	
I am concerned about the disapproval of others if I beat my horse. α (14)	Agree		Don’t know or mixed feelings	Disagree		
	A lot	A little	n/a	A lot	A little	
The weather will influence my horses’ health more than any management decisions I make. β (15)	Agree		Don’t know or mixed feelings	Disagree		
	A lot	A little	n/a	A lot	A little	
Spending time training my horse is worthwhile because it leads to better interactions between my horse and me.* (16)	Agree		Don’t know or mixed feelings	Disagree		
	A lot	A little	n/a	A lot	A little	

Statement	Participant's answer					Notes
Horses are more like machines, i.e. mechanically responding to instinctive urges without awareness of what they are doing. ∞ (17)	Agree		Don't know or mixed feelings	Disagree		
	A lot	A little	n/a	A lot	A little	
Animal welfare is a concept imposed on us by westerners. α (18)	Agree		Don't know or mixed feelings	Disagree		
	A lot	A little	n/a	A lot	A little	
I do not have time to groom my horse. β (19)	Agree		Don't know or mixed feelings	Disagree		
	A lot	A little	n/a	A lot	A little	
Horses are able to think to some extent to solve problems and make decisions about what to do. ∞ (20)	Agree		Don't know or mixed feelings	Disagree		
	A lot	A little	n/a	A lot	A little	

Behavioural beliefs* 1, 5, 9, 13, 16.
Subjective norms α 2, 6, 10, 14, 18.
Perceived behavioural control β 3, 7, 11, 15, 19.
Belief in animal minds ∞ 4, 8, 12, 17, 20.
