# Supplementary material for: Working horse welfare in Senegal is linked to owner’s socioeconomic status, their attitudes and belief in horse sentience
Source: PLoS One. 2024 Oct 18;19(10):e0309149. doi: 10.1371/journal.pone.0309149 (PMC11488707; doi:10.1371/journal.pone.0309149)
Supplement: S3 Table — (PDF) [file pone.0309149.s003.pdf]

S3 Table. Socioeconomic Status and Coverage of Needs tools

Instructions for the socioeconomic status tool

Please use the codes provided for each question.

Please explain the procedure to the participant as outlined below.

I would now like to ask you some questions about your household and living standards. If I ask you any question you don't want to answer, just let me know and I will go on to the next question or you can stop the interview at any time.

**Characteristics of the household (people who regularly live with the participant)**						
Family Members:<br>Please start with the head of the household/participant	Sex	Age group<br>1. 0-5 yrs.<br>2. 6-15<br>3. 16-59<br>4. 60+	Literacy Level<br>*Illiterate………..0*<br>*Literate…………..1*	Level of education<br>*Less than primary school…………6*<br>*Primary school……………………….1*<br>*Secondary School …………………...2*<br>*Higher………………………………….12*<br>*Don't know…………………………..98*<br>*Family member under 5 years..99*	Years of education<br>Number of years of education to date.<br>*Less than 1 year completed…………..00*<br>*1 or more years: record how many.*<br>*Don't know…………………………………….98*<br>*Family member under 5 years……...99*	Currently in school?<br>*Yes…………………………….1*<br>*No……………………………..2*<br>*Don't know………………..98*<br>*Member <5 or >24yrs…99*
1. Head of household						
2.						
3.						
4.						
5.						
6.						
7.						
8.						

Characteristics of the house and facilities			
**House Flooring**	Natural floor<br>Earth/sand……11<br>Dung……12<br><br>Other (*specify*)__________ 96	Rudimentary floor<br>Wood planks……21<br>Palm/bamboo……22	Finished floor<br>Parquet or polished wood……31<br>Vinyl or asphalt strips……32<br>Ceramic tiles……33<br>Cement……34<br>Carpet……35
**House Walls**	Natural walls<br>No walls……11<br>Cane/palm/trunks……12<br>Dirt……13<br><br>Other (*specify*)__________ 96	Rudimentary walls<br>Bamboo with mud…… 21<br>Stone with mud…… 22<br>Uncovered adobe…… 23<br>Plywood…… 24<br>Cardboard… …… 25<br>Reused wood…… 26	Finished walls<br>Cement……31<br>Stone with lime/cement ……32<br>Bricks… …… 33<br>Cement blocks …… 34<br>Covered adobe…… 35<br>Wood planks/shingles… …… 36
**House Roofing**	Natural roofing<br>No roof…… 11<br>Thatch/palm/leaf…… 12<br>Sod… …… 13<br><br>Other (*specify*)__________ 96	Rudimentary roofing<br>Rustic mat …… 21<br>Palm/bamboo …… 22<br>Wood planks… …… 23<br>Cardboard……24	Finished roofing<br>Metal……31<br>Wood……32<br>Calamine/cement fiber……33<br>Ceramic tiles……34<br>Cement ……35<br>Roofing shingles… ……36
**Sanitation** *(usual toilet facility)*	Flush / pour flush<br>Flush to piped sewer system……11<br>Flush to septic tank……12<br>Flush to pit (latrine)……13<br>Flush to somewhere else……14<br>Flush to unknown place……15	Pit Latrine<br>Ventilated Improved Pit latrine (VIP)……21<br>Pit latrine with slab……22<br>Pit latrine without slab / open pit……23<br><br>Composting toilet …… 31	Bucket …… 41<br>Hanging toilet/hanging latrine……51<br>No facilities or bush or field……61<br><br>Other (*specify*)__________ 96
**Sanitation: Sharing Facility**	*Do you share this toilet facility with other households?*	Yes……1<br>No……2	
**Cooking Fuel**	Electricity …… 01<br>Liquid Propane Gas (LPG)……02<br>Natural gas…… 03<br>Biogas…… 04<br>Kerosene…… 05<br>Coal / Lignite…… 06	Charcoal……07<br>Wood……08<br>Straw/shrubs/grass……09<br>Agricultural crop …… 10<br>Animal dung……11	No Food Cooked in Household……95<br><br>Other (*specify*)__________ 96

Characteristics of the house and facilities			
**Primary Source of Drinking Water**	Piped water<br>Piped into dwelling...............................11<br>Piped into yard or plot..........................12<br><br>Public tap/standpipe ....13<br>Tubewell/borehole .... 21<br><br>Other (specify)_____________________ 96	Dug well<br>Protected well....................................... 31<br>Unprotected well .... 51<br>Water from spring<br>Protected spring .... 41<br>Unprotected spring 52	Rainwater .... 42<br>Tanker-truck .... 61<br>Cart with small tank/drum… .... 71<br>Surface water (e.g. river, dam, pond, canal)…81<br>Bottled Water 91
**Primary Source of Water: Distance to Water Source**	How long does it take to get to the water source, get water and come back?	Minutes (specify) _______________________<br>Water on Premises…………………………………00<br>Don't Know……………………………………………998	

**Assets:** which items does the household own?					
Electricity	Yes…………….1	No…………….2	Bicycle	Yes…………….1	No…………….2
Radio	Yes…………….1	No…………….2	Motorbike/Scooter	Yes…………….1	No…………….2
Refrigerator	Yes…………….1	No…………….2	Car or truck	Yes…………….1	No…………….2
Television	Yes…………….1	No…………….2	Animal-drawn Cart	Yes…………….1	No…………….2
Telephone (mobile or fixed)	Yes…………….1	No…………….2	Computer	Yes…………….1	No…………….2

INCOME MEASURES	
**Sources and amounts of monetary income**	
Sources	Amounts
• Salaries<br>• Retirement pensions<br>• Trade activities income<br>• Transport activities income<br>• Revenues from transfer (national)<br>• Revenues from transfer (international)<br>• Others	

**Coverage of family needs**
To what extent family needs (food, clothing, education, etc.) are covered by family income<br><br>1. Family income does not cover the family needs and have high debts, unable to pay in due time, stress and instability for the family<br>2. Family income does not cover the family needs and have low/moderate debt that can be stressful sometimes<br>3. Family income does not cover the family needs but no debts<br>4. The needs are partially covered<br>5. Family income covers needs but can’t save<br>6. Family income exceeds needs and can have savings, but not regularly (low /moderate saving)<br>7. Family income exceeds needs and can regularly save so that extra needs are supported whenever necessary (high saving)

Nom de l'énumérateur: Date
