# Supplementary material for: Working horse welfare in Senegal is linked to owner’s socioeconomic status, their attitudes and belief in horse sentience
Source: PLoS One. 2024 Oct 18;19(10):e0309149. doi: 10.1371/journal.pone.0309149 (PMC11488707; doi:10.1371/journal.pone.0309149)
Supplement: S4 Table — (PDF) [file pone.0309149.s004.pdf]

S4 Table: Animal Welfare Assessment: Primary Assessment of Welfare Experiences' (PAWE) Tool and Standardised Equine Based Welfare Assessment Tool (SEBWAT) measures

Instructions: Please make a cross on the analogue scale and explain in the notes why this place on the line was chosen.

DOMAIN	NEGATIVE EXPERIENCES	GUIDELINES	DEGREE & NOTES
NUTRITION	**Hunger**	Is the animal's BCS <2.5? Does the animal show signs of active food procurement or apathy? Does the animal have to compete with other animals for access to food?	**Notes:**
	**Thirst**	Does the animal present signs of thirst? How dehydrated is the animal? Is the access to water limited? Does the animal have to compete with other animals for access to water?	**Notes:**
	**Malnutrition**	Is the available food inappropriate or insufficient for the animal's species and size, and number of animals sharing resource? Is the food of poor quality?	**Notes:**
	**Pain – Intensity**	Does the animal present symptoms of gastroinstestinal pain and discomfort (e.g. bloating, flatulence, scraping flank/belly)? How much pain does the animal seem to be in?	**Notes:**

	**Pain – Frequency/ Length**	How often do you think the animal experiences this gastrointestinal pain? Is it short-lived or chronic? Is it extreme pain? Does it stop the animal from exhibiting normal behaviour?	**Notes**:

DOMAIN	POSITIVE EXPERIENCES	GUIDELINES	DEGREE & NOTES
NUTRITION	**Satiety**	Does the animal show signs of fullness and satisfaction? Is the animal's BCS between 2.5 and 3.5? Does the animal have access to and seem to be able to eat appropriate amounts of food?	**Notes:**
	**Quenched**	Does the animal possess free access to water? Is water at appropriate place and height? Is water source clean? Is it appropriate for the number of animals? Is the water source sheltered from the sun or exposed?	**Notes:**

	**Pleasure**	Does the animal have access to more than one kind of food with different textures and/or taste? I.e. is there variation present in the diet?	**Notes:**
	**Comfort**	Does the animal seem to have free access to food and water? Is the animal's movement uninhibited and unrestricted?	**Notes:**

DOMAIN	NEGATIVE EXPERIENCES	GUIDELINES	DEGREE & NOTES

ENVIRONMENT	**Discomfort**	Is the animal exposed to the weather or harsh light? Is there a lack of resting space? Is the resting space close to the area for avoiding excrement? How often does the animal's place seem to be cleaned? Is the animal's area generally unpleasant or uncomfortable? Is the substrate unsuitable?	**<u>Notes:</u>**
	**Loneliness**	Is the animal isolated from others of its kind? Does the animal have company when not working? Are there other animals from other species around with whom they interact? Are they being bullied by, or having to compete for resources with other animals?	**<u>Notes:</u>**
	**Constraint**	How small is the shelter? Is the animal coerced into small spaces? Does it look oppressed? Can they move freely or is there restricted movement? Is the animal's movement restricted due to hobbling, tethering? Are multiple animals tied down together? Is there enough space for all the animals that use that shelter to enter, stand, or lie down together comfortably and without conflict?	**<u>Notes:</u>**

DOMAIN	POSITIVE EXPERIENCES	GUIDELINES	DEGREE & NOTES
ENVIRONMENT	**Comfort**	Does the animal have shelter from sun/rain/cold? Is the environment clean from garbage and parasites? Does the animal have appropriate bedding and resting area (dry, with appropriate amount and space), and an area for avoiding excrement? Is there a source of fresh air?	**Notes:**
	**Freedom**	How much space is there available for the animal to choose from? Can they move around freely? Are they free to move to a different area? Can they have active rewarding behaviours? Can they have access to preferred sites for resting, eating, and avoiding excrement? Is the animal free from tethers or hobbling?	**Notes:**
	**Sociability**	Is the animal housed alone or with other animals? Are they from the same or different species? Can the horse see other horses? Can they interact and touch each other? Can they choose whether to be with or away from them?	**Notes:**

DOMAIN	NEGATIVE EXPERIENCES	GUIDELINES	DEGREE & NOTES
HEALTH	**Discomfort**	Does the animal present lesions, injuries, symptoms of infectious diseases, lameness or **neglect** (i.e. are there signs of lack of provision of professiona/adequate medical care?Is it showing any signs of breathing difficulty or negative vocalisation? Is it weak or showing signs of exhaustion? Does the animal have unsuitable footing? Is there any sign of mutilations?	**Notes:**
	**Pain - intensity**	Does the animal show symptoms of significant acute or chronic pain caused by traumatic injury or pathological processes? Does this pain lead to loss or decrease in mobility? Are there open lesions?	**Notes:**
	**Pain – Frequency/ Length**	How often do you think the animal experiences this pain due to trauma or disease? Is it short-lived or chronic? Is it extreme pain? Does it stop the animal from exhibiting normal behaviour?	**Notes:**

DOMAIN	POSITIVE EXPERIENCES	GUIDELINES	DEGREE & NOTES
HEALTH	**Comfort**	Is the animal fit and healthy/uninjured? Does it seem comfortable and showing adequate levels of mobility and natural behaviour? Is there an absence or only small signs of injuries, disease,	**Notes:**

		or dysfunctional body systems? Are the animal's hooves healthy?	
	**Vitality**	Does the animal seem free from illnesses, and with vigour at a good fitness level? Does the animal seem well-rested?	**Notes:**

DOMAIN	NEGATIVE EXPERIENCES	GUIDELINES	DEGREE & NOTES
BEHAVIOUR	**Frustration**	Does the animal show signs of repetitive behaviour, increased reactiveness or hypersensitivity? Is the animal prevented from exercising natural sexual behaviour?	**Notes:**
	**Depression**	Does the animal show signs of helplessness or of having 'given up'? Is the animal exhausted or showing signs of apathy and lack of behavioural expression? (Disinterested, unresponsive). Is the animal showing signs of boredom or confusion? Are there signs of very low positive stimulation (barren environment, lack of contact)?	**Notes:**
	**Anxiety**	Does the animal present increased respiratory activity? Is there clear avoidance behaviour from human interaction or from other animals? Is it	**Notes:**

		alarmed/tense with moving ears and tension in the face and body? **HUMAN APPROACH**	
	**Anger**	Is the animal manifesting aggression and rage? Does it require restraint for allowing approach? Does the animal present ears flat-back against the head, or intention to harm (e.g. turning hindquarters towards object of aggression, Flared nostrils, white of the eye, tense muzzle, ears flat back or partially back, triangulation of eye, head held high and upright, body tense)? **HUMAN APPROACH**	**Notes:**
	**Panic**	Is the animal nervous and seeking escape routes? Is it actively and aggressively avoiding other animals and humans? Does it show extreme signs of distress **and fear**? **HUMAN APPROACH**	**Notes:**
**DOMAIN**	**POSITIVE EXPERIENCES**	**GUIDELINES**	**DEGREE & NOTES**
**BEHAVIOUR**	**Confidence**	Does the animal fully explore its environment and exhibit natural and rewarding behaviour? Is their behaviour mostly unchanged by the approximation by assessors/other people? Is the animal in control? Is it eager to approach handler in an alert, relaxed manner?	**Notes:**

	Security	Does the animal show signs of presenting active approaching or curiosity? Can they back away if they want to? **HUMAN APPROACH**	**Notes:**
	Calmness	Is the animal calm? Is it relaxed when observed from a distance? After animal notices assessors, does it continue to act normally? Does it allow the owner and other people to approach it? Can it be touched without showing signs of fear? **HA**	**Notes:**
	Affection	Is the animal sociable? If around other animals, are they clearly fond of any of them? What about to humans (owner and assessors)? Are there signs of positive reinforcement between handler and animal (praise/stroking)? Is the animal receptive? Do they seek affection by approaching either humans or other animals?**HA**	**Notes:**
	Joy	Is the animal interested in and curious about their surroundings (e.g. ears alert or one partially back)? Do they show active interest in nearby animals and humans? Do they look energized? Are they showing signs of play?	**Notes:**
	Choice	Can the animal exhibit their normal behaviours? Does it have choice to interact with certain individuals whilst avoiding others? Can they interact and display sexual behaviours?	**Notes:**

Selected SEBWAT Measures

**Body Condition:**	1: Very thin		2: Thin	3. Medium	4: Fat	5: Very fat
**Gait:**	0: Not compromised		1: Moderately compromised	2: Highly compromised		3: Unable to bear weight
**Lesions:**	**Severity**	0: None		1: Superficial or healed	2: Open	3: Deep
	**Size**	0: None		1: Small (4-16cm^2)	2: Medium (17-64cm^2)	3: Large (>64cm^2)
**General Health Status (give an overall, holistic view):**			1: Good	2: Fair		3: Poor
